# Evaluation of Genetic Markers as Instruments for Mendelian Randomization Studies on Vitamin D

**DOI:** 10.1371/journal.pone.0037465

**Published:** 2012-05-21

**Authors:** Diane J. Berry, Karani S. Vimaleswaran, John C. Whittaker, Aroon D. Hingorani, Elina Hyppönen

**Affiliations:** 1 Centre for Paediatric Epidemiology and Biostatistics and MRC Centre for the Epidemiology of Child Health, UCL Institute of Child Health, London, United Kingdom; 2 Department of Epidemiology and Population Health, London School of Hygiene and Tropical Medicine, London, United Kingdom; 3 Genetic Epidemiology Group, Department of Epidemiology and Public Health and the Centre for Clinical Pharmacology, University College London, London, United Kingdom; Instituto de Higiene e Medicina Tropical, Portugal

## Abstract

**Background:**

Mendelian randomization (MR) studies use genetic variants mimicking the influence of a modifiable exposure to assess and quantify a causal association with an outcome, with an aim to avoid problems with confounding and reverse causality affecting other types of observational studies.

**Aim:**

We evaluated genetic markers that index differences in 25-hydroxyvitamin D (25(OH)D) as instruments for MR studies on vitamin D.

**Methods and Findings:**

We used data from up-to 6,877 participants in the 1958 British birth cohort with information on genetic markers and 25(OH)D. As potential instruments, we selected 20 single nucleotide polymorphisms (SNP) which are located in the vitamin D metabolism pathway or affect skin pigmentation/tanning, including 4 SNPs from genome-wide association (GWA) meta-analyses on 25(OH)D. We analyzed SNP associations with 25(OH)D and evaluated the use of allele scores dividing genes to those affecting 25(OH)D synthesis (*DHCR7, CYP2R1*) and metabolism (*GC, CYP24A1, CYP27B1*). In addition to the GWA SNPs, only two SNPs (*CYP27B1*, *OCA2*) showed evidence for association with 25(OH)D, with the *OCA2* association abolished after lifestyle adjustment. Per allele differences varied between −0.02 and −0.08 nmol/L (*P*≤0.02 for all), with a 6.1 nmol/L and a 10.2 nmol/L difference in 25(OH)D between individuals with highest compared lowest number of risk alleles in synthesis and metabolism allele scores, respectively. Individual SNPs but not allele scores showed associations with lifestyle factors. An exception was geographical region which was associated with synthesis score. Illustrative power calculations (80% power, 5% alpha) suggest that approximately 80,000 participants are required to establish a causal effect of vitamin D on blood pressure using the synthesis allele score.

**Conclusions:**

Combining SNPs into allele scores provides a more powerful instrument for MR analysis than a single SNP in isolation. Population stratification and the potential for pleiotropic effects need to be considered in MR studies on vitamin D.

## Introduction

There has been much interest in the potential effects of vitamin D on a wide range of health outcomes, and vitamin D deficiency has been suggested to predispose to common chronic diseases such as diabetes, cancer and cardiovascular disease [1]–[6]. Effects on bone health are undisputed and severe vitamin D deficiency leads to rickets in children and osteomalacia in adults [7]. However, the recent report by the Institute of Medicine on vitamin D, concluded that “with the exception of measures related to bone health, the potential indicators examined are currently not supported by evidence that could be judged either convincing or adequate in terms of cause and effect, or informative regarding dose–response relationships for determining nutrient requirements.” [8]. This reflects the fact that much of the evidence has been obtained from observational studies, with only a handful of randomized controlled trials evaluating the effects of vitamin D supplementation.

Observational associations of 25-hydroxyvitamin D concentration (25(OH)D, a marker for nutritional vitamin D status) and adverse health outcomes may provide evidence of a causal link, but could also arise from limitations with this type of study. For example, studies on vitamin D may be prone to confounding, as status is associated with risk factors for chronic diseases such as obesity [9]. Reverse causality may be a problem as given the strong role of sunlight induced skin synthesis in vitamin D production, lower concentrations of 25(OH)D could be a consequence of less time spent outdoors and hence, caused by an underlying disease rather than being the cause of it. Mendelian randomization (MR) analysis refers to the use of genetic variants that index the exposure of interest (in this case vitamin D intake/status) to gain insight on whether the relationship between an exposure and disease is causal [10], [11]. If lower vitamin D status is causally related to an adverse outcome (e.g. cardiovascular disease), a genetic variant associated with lower 25(OH)D concentration should be associated with a higher risk of cardiovascular disease (in relation to its effect on 25(OH)D). The genetic association, unlike the directly observed association of vitamin D intake/status itself, will be less prone to confounding (as the genotype is assigned at random) and free from reverse causation since the genotype is not modifiable by disease [12].

Nevertheless, the MR approach has some limitations [12]–[14]. An assumption of MR analysis is that the effect of a genetic variant on an outcome functions only via the intermediate exposure, such as a lifestyle factor or biomarker. However, a genetic variant may result in multiple biological alterations (pleiotropy). Hence, if these alterations also independently affect relevant outcomes not via of the intermediate phenotype, this may lead to associations through a mechanism that does not involve the exposure of interest [10]. Genetic confounding may also result in violation of MR assumptions if closely located genetic variants are inherited together (i.e. in the presence of linkage disequilibrium) and affect the outcome outside of the mechanism of the exposure, or if there is evidence for population stratification [10].

In this study, we used information from the large nationwide survey of 1958 British birth cohort to evaluate genetic markers for the use as instruments in MR studies on vitamin D.

## Methods

### Ethics statement

Ethical approval for the biomedical survey was given by the South-East Multi-Centre Research Ethics Committee. Written informed consent for the use of information in medical studies was obtained from the participants.

### Participants

Detailed description of the study has been published previously [15]. In brief, participants are from the 1958 British birth cohort (1958BC), initially including all births in England, Scotland or Wales during one week in March 1958 (n = 17,638) [15]. Between September 2002 and June 2004, 11,971 participants still residing in Britain were invited to participate in a biomedical survey. At the time of data collection participants were aged 44–46 years old, and 9,377 (78%) completed at least one questionnaire and 7,591 (81% of the respondents) had valid 25(OH)D measures. The 1958BC is almost entirely a white European population (98%) [16], and since we utilize genetic data for these analyses, 158 individuals of other ethnic groups and one pregnant participant were excluded. Data was further restricted by availability of genetic data with 4,572–6,877 individuals included in the single nucleotide polymorphism (SNP) analyses. Analyses using the vitamin D allele score were done in participants with full data on 25(OH)D synthesis (n = 5,623) and/or metabolism (n = 5,856) markers (for full description, please see below).

### Measurement of biochemical and clinical parameters

Serum 25(OH)D concentrations were measured using an automated IDS OCTEIA enzyme-linked immunosorbent assay (ELISA) (Dade-Behring BEP2000 analyzer), standardized according to the mean from Vitamin D External Quality Assessment Scheme (DEQAS) [17]. Serum IGF-1 concentration was measured using the Nichols Advantage IGF-1 chemiluminescence immunoassay (referenced against World Health Organization 1st International Reference Reagent 1988; IGF-1 87/518) and glycosylated hemoglobin (HbA1C) with high-performance liquid chromatography (as certified by the US National Glycohemoglobin Standardization Program (NGSP) [18]). Triglycerides and total and high-density lipoprotein (HDL) cholesterol were measured by standard autoanalyzer methodology and low-density lipoprotein (LDL) cholesterol was calculated using the Friedewald formula. Fibrinogen was determined by the Clauss method, D-dimer by ELISA assay and C-reactive protein (CRP) was assayed by nephelometry (Dade Behring). Von Willebrand factor (vWF) antigen was measured by Decollates ELISA and tissue plasminogen activator (tPA) antigen by Biopool elisa. Total IgE was assayed using the HYTEC automated enzyme immunoassay [19].

Weight, height and waist circumference were measured at 45 years of age. Blood pressure was determined as an average of three repeated measures (Omron 705CP automated sphygmomanometer). For forced expiratory volume 1 (FEV_1_) and forced vital capacity (FVC), the highest technically satisfactory values (three repeated measures) were used [20].

### Social, dietary and lifestyle factors

Socioeconomic position was assessed using the Registrar General's occupational classification categorized as I and II (managerial and professional), III (non-manual), III (manual), and IV and V (manual unskilled) [21]. Individuals who were institutionalized, retired, or long-term unemployed were classified separately. Physical activity was determined as recreation Metabolic Equivalent of Task (MET) hours, derived from reported frequencies and usual durations for up to 37 activities [22]. Smoking was recorded as never/ex-smoker vs current smoker based on smoking history recorded at ages 23, 33, and 42 years. Frequency and amounts of alcohol consumption were reported at 45 years. Information on current geographical region of residence was based on Government Office Regions, and categorized as South (South East, South West, and Greater London), Middle (East Anglia, Midlands, and Wales), North (North, North West, and Yorkshire and the Humber), and Scotland. Geographical region was dichotomized when used as an outcome (South/Middle vs North/Scotland).

The following factors (measured at 45 years) were also considered and dichotomized: time spent watching a television/using a PC (coded as <1 h vs ≥1 h), time spent outside (coded as <1 h vs ≥1 h), protecting skin in the sun (often/sometimes vs rarely/never), oily fish consumption (≥3 days/week vs. 3 days/week), use of vitamin D supplement, and season of blood drawn (winter/spring vs. summer/autumn).

### Selection of candidate genes and SNPs

**Figure 1:**
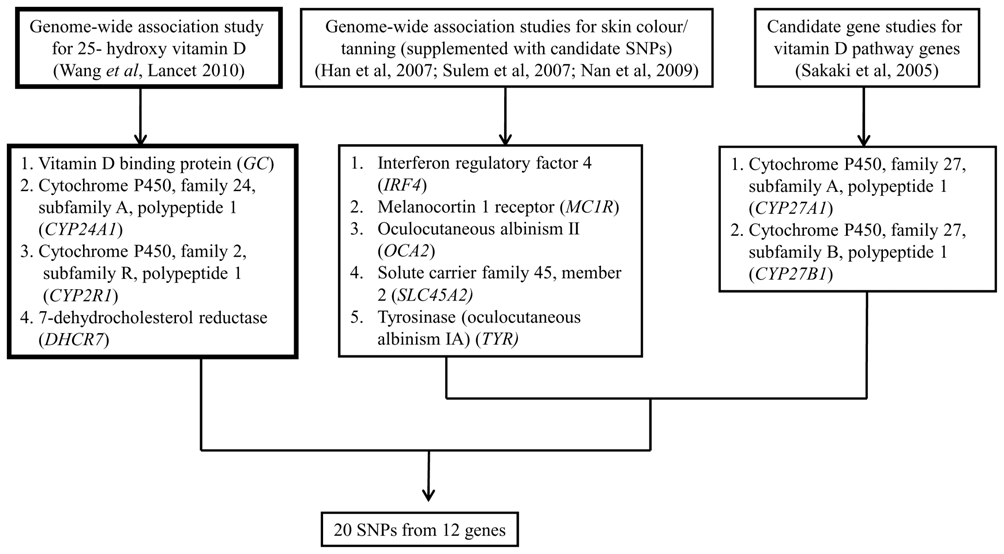
Strategy for SNP selection using genome-wide association and candidate gene studies.

We selected 20 SNPs from 12 genes, which are involved in the vitamin D pathway or affect skin pigmentation or the ability to tan [23]–[26]. Four of the SNPs were identified as hits in the recently published genome-wide association (GWA) meta-analysis for vitamin D insufficiency (n∼34,000, including the 1958 British birth cohort) [23] (**Figure 1**). The GWA *GC* SNP rs2282679 was in high linkage disequilibrium with the candidate *GC* SNP rs4588 (r^2^ = 0.98) and hence due to higher numbers with data available (n = 6,551 vs. 5,224), we have chosen rs4588 as a proxy for the SNP rs2282679 in the present study. Two of the SNPs were identified as candidates based on the available evidence from the literature pertaining to their potential roles in vitamin D metabolic pathway (**Figure 1**). Fourteen SNPs were chosen based on the GWA and candidate studies for skin colour/tanning. A complete list of the selected genes/SNPs is shown in the **Table S1**.

### Genotyping

The SNPs (rs4588, rs12785878, rs10741657, rs6013897, rs10877012, rs17470271, rs7495174, rs4778241, rs4778138, rs13289, rs1805005, rs2228479) were genotyped using the Taqman platform (Applied Biosystems, Warrington, UK) [27]. The custom genotype SNPs had passed the inclusion criteria (Hardy-Weinberg Equilibrium (HWE) *P* value >0.01 [28], MAF>0.01 and call rate >80%) (**Table S1**). The remaining SNPs were genotyped on the platforms of Affymetrix 6.0 and Illumina 550 K Infinium by the two sub-studies of WTCCC2 and TIDGC and QC procedures were applied accordingly [29], [30]. Imputation of SNPs genotyped by the WTCCC2 and TIDGC was done using the software Impute [31] (as outlined for the 1958BC in the GWAS on 25(OH)D [23]). In the TIDGC sub-study, two SNPs (rs16891982, rs1805007) were imputed, and in the WTCCC2, three SNPs (rs16891982, rs11648785, rs464349) were imputed (a call rate threshold of 0.9 was used for the imputed SNPs).

### Statistical methods

Natural log transformation was used for 25(OH)D and the biomarkers (except for lung function) to improve the approximation of the normal distribution. Distributions were assessed before and after transformation using quantile-normal plots. Variation in continuous outcomes was evaluated by linear regression and, in dichotomous outcomes, by logistic regression with the p-values from Wald tests.

The four SNPs in genes with confirmed associations with 25(OH)D (*GC*; rs4588, *CYP2R1*; rs10741657, *DHCR7*; rs12785878, *CYP24A1*; rs6013897) [23] were considered as possible instruments by default, and taken forward to subsequent analyses. For the other genes, we tested their associations with 25(OH)D using linear regression, taking forward all SNPs with a p-value below the Bonferroni corrected threshold of <0.007 (≤0.05/number of candidate genes) to control for multiple testing. The number of candidate genes (rather than number of SNPs) was used in Bonferroni correction to account for moderate linkage disequilibrium between SNPs. To indicate the strength of the SNP as instrument we included the F-statistic from a simple linear regression model with 25(OH)D. The F-statistic in a simple linear regression model is derived from the proportion of the variation explained by the genetic variant in the phenotype given the sample size [32]. As a rule of thumb an F-statistic less than 10 is taken to indicate a weak instrument [33]. Formal MR analysis often uses instrumental variable (IV) regression by two-stage least squares estimator, however this may introduce a bias if sample size is small and there is too much variability in the estimated association between the SNP and intermediate phenotype [34]. The relative bias of IV analysis compared to ordinary least squares (linear regression) can be approximated as the inverse of the strength of the instrument (1/F-statistic) [34]. Interactions between the SNPs were tested by including the interaction terms in the linear regression model on 25(OH)D adjusting for sex with p-values corrected by the number of vitamin D SNPs to account for multiple testing (0.05/5, *P*<0.01).

Relatively small proportion of participants had missing data on confounders/covariates (15% with at least one missing value) after restriction by the availability of genetic information. All main analyses requiring confounder adjustments were run with complete information. To investigate whether the results were sensitive to missing information in covariates, multivariate imputation by chained equations was used to impute missing values [35] and the main analyses were re-run. The results were identical whether based on models run on complete data or on data obtained by multiple imputation. To assess whether the SNP associations with 25(OH)D were confounded, we adjusted for lifestyle and social factors in the linear regression model examining the associations of the SNPs with 25(OH)D (namely time spent outside, sun cover, oily fish consumption, vitamin D supplements, season, smoking, alcohol consumption, PC/TV time, recreational metabolic equivalent task (MET) hours, social class, body mass index (BMI), abdominal obesity, geographical region, and sex). We also analysed the direct associations between the SNPs and social, dietary and lifestyle factors using logistic regression. Interactions between the SNPs and lifestyle/social factors were evaluated by including appropriate interaction terms in the model. In these analyses a Bonferroni correction was used to correct for the number of factors tested, with the p-value threshold determined as 0.05/14 (*P*<0.004).

To investigate whether the relationship between the SNPs and 25(OH)D was due to other health factors and differing genetic pathways (“genetic confounding”), we adjusted for the available biomarkers (von Willebrand factor, tPA, D-dimer, fibrinogen, CRP, IgE, triglycerides, low density lipoproteins, high density lipoproteins and total cholesterol, FEV, diastolic and systolic blood pressures, IGF1 and HbA1c) in the linear regression model examining the genetic marker association with 25(OH)D. We also investigated the direct SNP- biomarker associations adjusted for 25(OH)D, with the assumption that if pleiotropy did exist these associations should appear fairly strong, and not be affected by 25(OH)D adjustment. Interactions between 25(OH)D and SNPs with biomarkers were also tested. Bonferroni corrected *P*-value for these analyses was <0.003 (0.05/15, where the denominator is the number of biomarker tests for each SNP).

**Figure 2:**
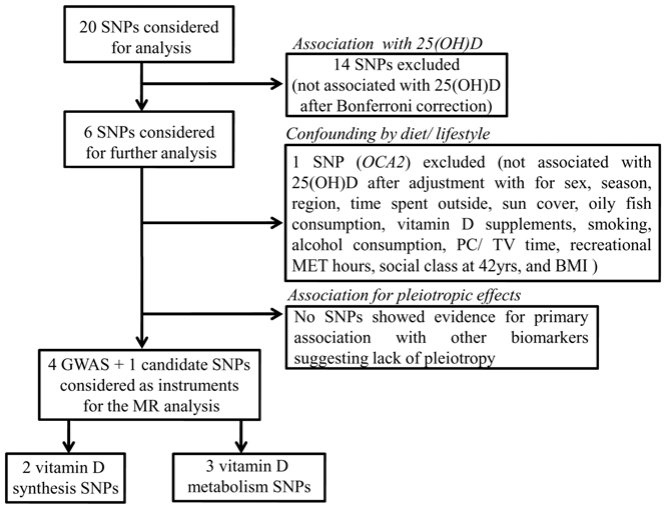
Genetic variation in the vitamin D synthesis and metabolic pathway. Skin exposure to ultraviolet B (UVB) radiation initiates the conversion of 7-dehydrocholesterol to previtamin D3. 7-dehydrocholesterol reductase (***DHCR7***) encodes the enzyme 7-dehydrocholesterol reductase, which converts 7-dehydrocholesterol to cholesterol, thereby removing the substrate from the synthetic pathway of vitamin D3. The previtamin D3 in turn gets converted to vitamin D3 in a heat-dependent process. Vitamin D (represents D2 or D3) is transported to the liver, where it is converted by vitamin D-25-hydroxylase (***CYP2R1***) to 25-hydroxyvitamin D [25(OH)D]. This is the major circulating form of vitamin D that is used by clinicians to determine vitamin D status. This form of vitamin D is biologically inactive; it is bound to the vitamin D-binding protein (***GC***), transported to the kidneys and converted by 25-hydroxyvitamin D-1α- hydroxylase (1-OHase) (***CYP27B1***) to the biologically active form 1,25-dihydroxyvitamin D3 (Calcitriol). Calcitriol increases the expression of 25-hydroxyvitamin D-24- hydroxylase (24-OHase) (***CYP24A1***) to catabolise 25(OH)D to the water-soluble, biologically inactive calcitroic acid, which is excreted in the bile. *DHCR7* and *CYP2R1* function upstream of the production of 25(OH)D and hence, termed as 25(OH)D synthesis indicators, while *GC*, *CYP27B1* and *CYP24A1* function downstream of the 25(OH)D production and hence, termed as 25(OH)D metabolism indicators.

“Synthesis score” was created using the two SNPs in genes encoding proteins involved in 25(OH)D synthesis (*DHCR7* and *CYP2R1*), both of which had been identified through the GWA meta-analyses on 25(OH)D [23]. Three SNPs in genes encoding proteins involved in 25(OH)D metabolism (*GC, CYP24A1* and *CYP27B1*) were included in the “metabolism score”, with analyses repeated only including the GWA confirmed SNPs (“metabolism^GWA^ score”, *GC* and *CYP24A1*) (**Figure 2**). The allele score was created by counting the number of vitamin D lowering alleles. For alleles scores based on metabolizing genes (*GC*, *CYP24A1* and *CYP27B1*), there was only one subject with six lowering alleles, and 36 subjects with five lowering alleles, these groups were combined with those who had four lowering alleles. Likewise for alleles in genes involved in 25(OH)D synthesis (*CYP2R1* and *DHCR7*) score, only 97 subjects had four lowering alleles, so this group was combined with those who had three lowering alleles. As described above, we examined the associations of allelic score indicators with potential confounders and disease-relevant biomarkers.

We used simulation to carry out illustrative power calculations for the association between vitamin D and systolic blood pressure using single SNPs, separate allele scores and allele scores together as instruments (**Appendix S1**). We assumed effect sizes as observed in the 1958BC, notably a 5% reduction in blood pressure by each 10 nmol/l increase in 25(OH)D, and the observed SNPs/scores effects on 25(OH)D. Data was simulated 1,000 times for a given sample size and two-staged least squares regression was run. The parameter of interest was tested and power was estimated from the proportion of times the test *p*-value was less than the significant level α = 0.05. Analyses were carried out using STATA, version 11 [36].

## Results

**Figure 3:**
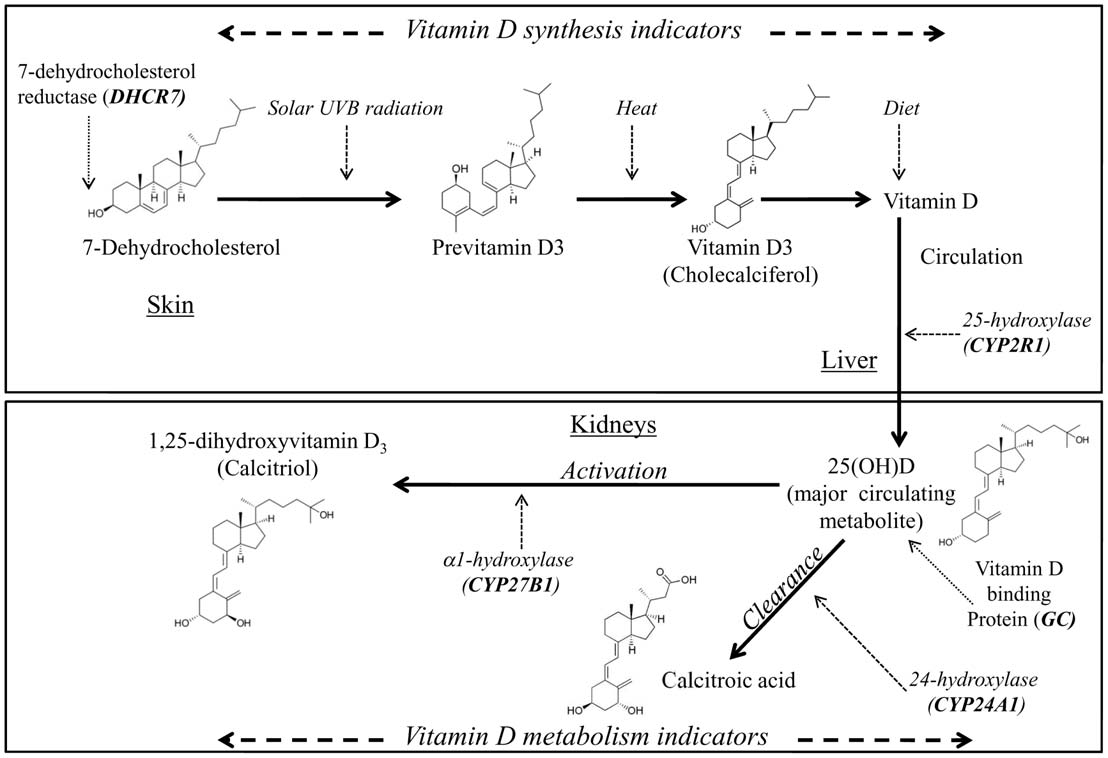
The selection of vitamin D SNPs for the use as instruments in Mendelian Randomization (MR) analysis.

The 4 SNPs that had been chosen on the basis of the 25(OH)D GWAS (in *GC*, *CYP2R1*, *DHCR7, CYP24A1*) [23] were associated with 25(OH)D in the 1958BC (*P*≤0.016). Of remaining 16 SNPS, one SNP in *OCA2* (rs7495174) had a significant association with 25(OH)D after Bonferroni correction (*P* = 0.002) (**Table 1**). These five SNPs were taken forward for further evaluation together with one SNP (*CYP27B1*, rs10877012, *P* = 0.008) (**Figure 3**) that fell slightly below the significance threshold, but had previous evidence for replication [37]. There was no evidence for SNP-SNP interactions between any of the six SNPs (*P* for all comparisons ≥0.08, data not presented).

**Table 1:** Association of SNP with ln 25-hydroxyvitamin D adjusted for sex.

Gene	SNP	*n*	MAF	Beta for minor allele	SE	*P* value	% of variance explained	F-statistic	Relative bias†, %
***GWA on 25(OH)D***									
GC	rs4588	6027	0.30	−0.08	0.009	**1.48×10^−17^**	1.18	73.4	1.4
*DHCR7/NADSYN1*	rs12785878	6504	0.22	−0.05	0.009	**1.2×10^−6^**	0.35	22.4	4.4
*CYP2R1*	rs10741657	5968	0.40	0.03	0.009	**0.0003**	0.21	13.3	8.1
*CYP24A1*	rs6013897	6534	0.20	−0.03	0.010	0.016	0.07	5.4	21.1
***Vitamin D pathway genes***									
*CYP27B1*	rs10877012	6877	0.33	−0.02	0.008	**0.008**	0.09	7.2	16.8
*CYP27A1*	rs17470271	5831	0.44	0.005	0.009	0.55	-	0.4	-
***GWA on skin colour/tanning***									
*OCA2*	rs7495174	5013	0.06	−0.06	0.02	**0.002**	0.16	8.5	12.2
*OCA2*	rs4778241	4961	0.17	−0.01	0.01	0.39	-	0.6	-
*OCA2*	rs4778138	5036	0.12	−0.02	0.01	0.13	0.03	1.9	75.8
*OCA2*	rs12913832	4989	0.22	0.003	0.01	0.76	-	0.1	-
*SLC45A2*	rs13289	5039	0.38	−0.02	0.009	0.013	0.10	6.5	19.3
*SLC45A2*	rs16891982	4843	0.02	−0.03	0.04	0.48	-	0.5	-
*MC1R*	rs11648785	4816	0.30	−0.02	0.01	0.037	0.07	4.0	30.0
*MC1R*	rs1805005	5091	0.12	0.02	0.01	0.14	0.02	2.0	81.6
*MC1R*	rs464349	4966	0.46	−0.01	0.01	0.25	0.01	1.5	-
*MC1R*	rs2228479	5084	0.10	0.007	0.02	0.65	-	0.1	-
*MC1R*	rs1805007	4572	0.10	−0.003	0.02	0.87	-	0.0	-
*IRF4*	rs12203592	5184	0.22	−0.01	0.01	0.20	0.01	1.6	-
*IRF4*	rs12210050	4988	0.20	−0.01	0.01	0.58	-	0.3	-
*TYR*	rs1393350	4992	0.29	0.01	0.01	0.48	-	0.5	-

MAF, minor allele frequency.

† Relative bias has not been estimated where the SNP has an F-statistic less than 1.9.

**Figure 4:**
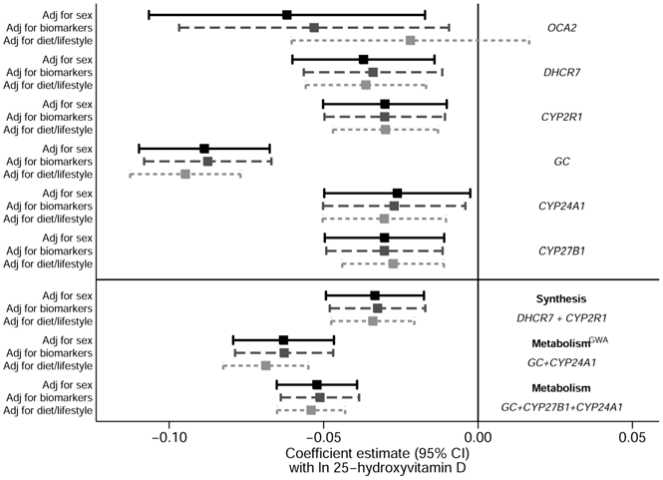
Association between the SNPs, synthesis, metabolism and metabolism^GWA^ allele scores and ln 25(OH)D with and without adjustment for biomarkers, dietary and lifestyle indicators. The bars are the 95% CI. *Biomarkers*: coagulation markers- von Willebrand factor, tPA and D-dimer; Inflammatory markers- fibrinogen and CRP; Lipid marker- Triglycerides, low density lipoproteins, high density lipoproteins and total cholesterol; Lung function marker- FEV; Cardiovascular disease related factors- diastolic and systolic blood pressures, IgE, IGF1 and HbA1c). *Dietary and lifestyle markers*: time spent outside, sun cover, oily fish consumption, vitamin D supplements, season, smoking, alcohol consumption, PC/TV time, recreational MET hours, social class, body mass index, abdominal obesity and geographical region.

We next examined whether the associations with 25(OH)D observed for these six SNPs, were sensitive to adjustment for geographical region, dietary and lifestyle factors or available biomarkers (**Figure 4**). The missing data for the lifestyle and dietary factors, and biomarkers ranged from 0.2% (BMI) to 7.7% (time spent outside). Most associations between the SNPs and 25(OH)D concentrations were not affected by these adjustments, however, an exception was *OCA2*, as its association was no longer present after adjustment for sex, season, geographical region, time spent outside, sun cover, oily fish consumption, vitamin D supplements, smoking, alcohol consumption, PC/TV time, recreational MET hours, social class at 42 yrs, and BMI (beta −0.06, *P* = 0.007 for unadjusted vs. −0.02, *P* = 0.27 adjusted, **Figure 4**). Due to the lack of independent association with 25(OH)D, *OCA2* was considered as unsuitable for the use as a proxy of 25(OH)D concentrations and removed from subsequent analyses. For the remaining five SNPs, we found no significant interactions by dietary and lifestyle factors on their influence on 25(OH)D (*P*>0.08 for all comparisons, data not presented). We also observed no evidence for pleiotropic effects for these SNPs, as no strong associations were observed with any of the available biomarkers with adjustment for 25(OH)D concentrations (**Table S2**). Furthermore, 25(OH)D did not modify the associations observed between the SNPs and the biomarkers (*P* interaction for all comparisons ≥0.07).

**Figure 5:**
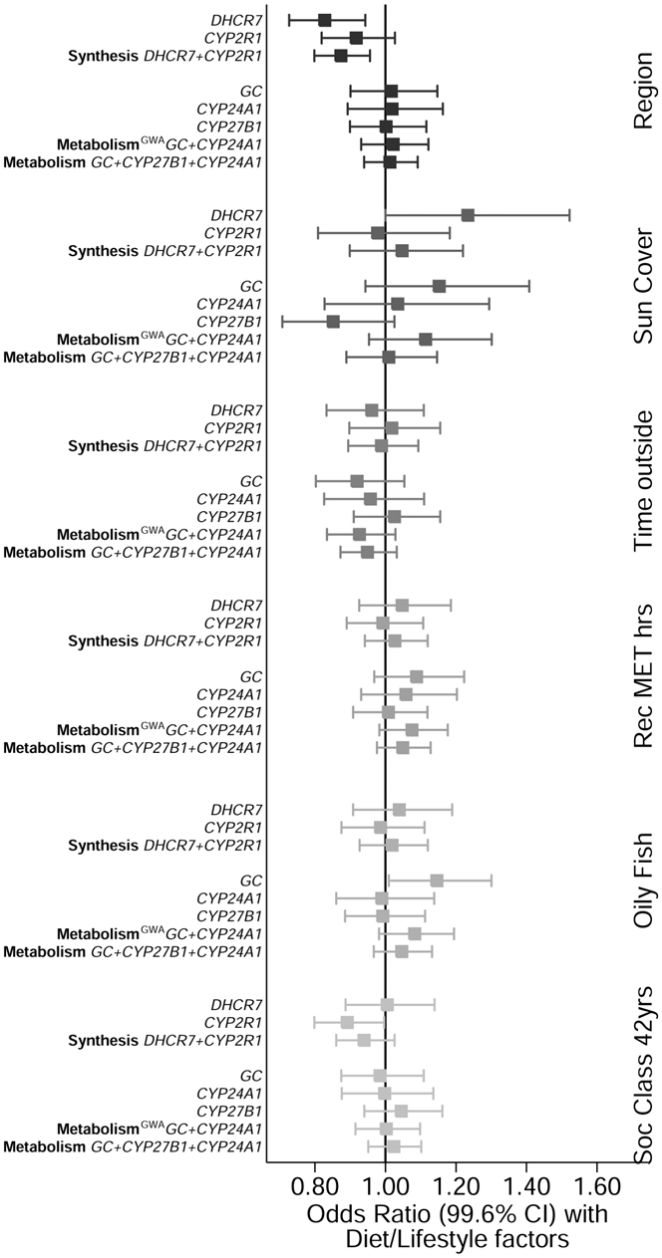
Associations of the five SNPs and allele scores with geographical region, social, dietary and lifestyle factors. The bars are the 99.6% CI. The effects of the allele scores and the individual SNPs for each lifestyle factor can be identified based on the intensity of the coloured boxes.

The combined 25(OH)D synthesis score (including SNPs in *DHCR7* and *CYP2R1*), explained 0.56% of the variation of 25(OH)D concentrations and there was a 6.1 nmol/l difference in 25(OH)D between top and bottom groups in the allelic score (**Table 2**). The allele score based on 25(OH)D metabolism GWAS SNPs (*GC* and *CYP24A1*) explained 1.04% of the variation of 25(OH)D concentrations and there was a 7.9 nmol/l difference in 25(OH)D between top and bottom groups in the allelic score, while the metabolism score also including *CYP27B1* explained 1.12% of the variation of 25(OH)D concentrations, with a 10.2 nmol/l difference between top and bottom quartile categories. As seen in **Figure 4**, associations between the allele scores and 25(OH)D were unaffected by adjustment for lifestyle and dietary factors or biomarkers. There were some associations between individual SNPs and dietary and lifestyle factors, notably rs10741657 from *CYP2R1* which was associated with social class (*P* = 0.003), and *GC* SNP rs4588 which was associated with oily fish consumption (*P* = 0.002), while allele scores were not associated with any of the lifestyle factors (**Figure 5**). However, there was an association between 25(OH)D synthesis allele score and geographical region even after applying Bonferroni correction (for the number of dietary and lifestyle factors tested), which reflected the strong association between *DHCR7* SNP rs12785878 with geographical region (*P* = 3.0×10^−5^). As with the single variants, we observed no evidence for pleiotropic effects as the allele scores were not associated with the biomarkers after adjustment for 25(OH)D (**Table S2**).

**Table 2:** Association of Allele Scores with ln 25-hydroxyvitamin D concentrations adjusted for sex.

	N	Geometric Mean (95% CI)	Beta	SE	*P* value	% of variance explained	F-statistic	Relative Bias, %
*Synthesis score* *								
0	587	56.2 (54.0, 58.5)	Reference	Reference	-	-	-	
1	2025	55.1 (54.0, 56.2)	−0.02	0.02	-	-	-	
2	2276	53.0 (52.1, 54.0)	−0.06	0.02	-	-	-	
3,4	968	50.2 (48.8, 51.6)	−0.11	0.02	-	-	-	
Synthesis score, per allele	5856	-	−0.04	0.007	6.1×10^−9^	0.56	33.4	3.1
*Metabolism* ^GWA^ score†								
0	1902	56.7 (55.5, 57.9)	Reference	Reference	-	-		
1	2489	53.2 (52.3, 54.2)	−0.06	0.01	-	-		
2	1305	50.3 (49.1, 51.5)	−0.12	0.02	-	-		
3, 4	240	48.8 (46.0, 51.7)	−0.15	0.03	-	-		
Metabolism^GWA^ score, per allele	5936	-	−0.06	0.007	1.8×10^−15^	1.04	63.1	1.6
*Metabolism score* ‡								
0	818	57.8 (56.0, 59.6)	Reference	Reference	-	-		
1	1836	55.6 (54.4, 56.8)	−0.04	0.02	-	-		
2	1767	52.6 (51.5, 53.7)	−0.09	0.02	-	-		
3	925	51.0 (49.5, 52.5)	−0.13	0.02	-	-		
4, 5, 6	277	47.6 (45.2, 50.2)	−0.19	0.03	-	-		
Metabolism score, per allele	5623	-	−0.05	0.006	8.8×10^−16^	1.12	64.6	1.6

* Synthesis SNPs include *DHCR7* and *CYP2R1*.

† Metabolism^GWA^ SNPs include *GC* and *CYP24A1*.

‡ Metabolism SNPs include *GC*, *CYP24A1* and *CYP27B1*.

**Figure 6:**
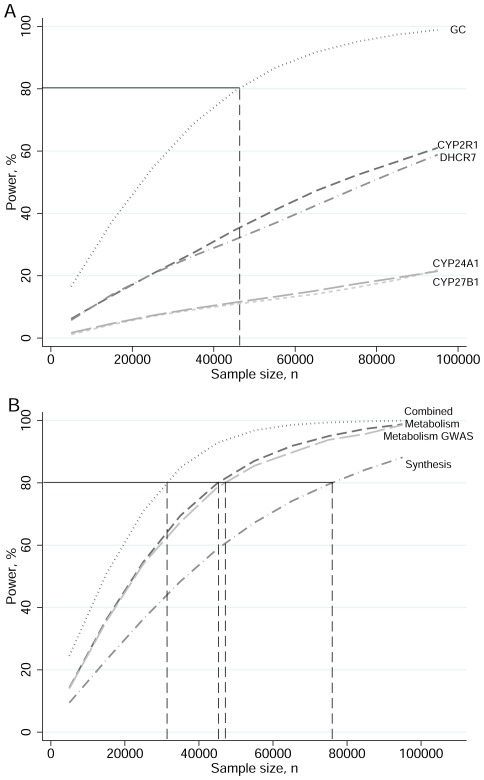
Power and sample size to detect the 5% decrease in blood pressure by 10 nmol/l increase in 25(OH)D observed in the 1958 British birth cohort using genetic proxy indicators (significance level α = 0.05). The curves in (**A**) from the bottom to the top of the graph are in the order of min effect size with *CYP27B1* (short dash), *CYP24A1* (long dash), *DHCR7* (dash dot), *CYP2R1* (dash), *GC* (dot). The curves in (**B**) from the bottom to the top of the graph are in the order of min effect size with Synthesis score (dash dot), Metabolism^GWA^ score (long dash) Metabolism score (dash), both scores (dot). The horizontal black line and attached vertical dashed lines indicate the sample size required for a study with 80% power using the genetic proxy.

Illustrative power calculations for a MR study on blood pressure using single SNPs, allele scores (synthesis, metabolism, metabolism^GWA^), and allele scores combined as instruments for vitamin D status are presented in **Figure 6A** & **6B**. Allele scores had greater power to detect an effect than the individual SNPs used in the scores. For example, to achieve 80% power using the synthesis allele score as instrument would require a sample size of 76,000 individuals, whilst using *CYP2R1* alone would require 155,000 individuals.

## Discussion

Insufficient intake of vitamin D has been proposed to affect up to 50% of the UK population [38], [39]. This is a potentially a very important public health issue because vitamin D (intake and/or deficiency) has been linked to several common diseases including cancer, diabetes, and cardiovascular diseases [40]. However, there is an urgent need to improve the evidence base for causal relations of vitamin D, as much of the evidence still relies on observational studies where it is difficult to disentangle causation from association. Randomized controlled trials are clearly the gold standard for demonstrating causality, however, they are expensive and time consuming. In this paper, we describe methodological work related to a complementary approach for causal analysis; notably, the rationale for identification and process to evaluate 25(OH)D associated SNPs as tools for future MR analysis of vitamin D.

We identified five SNPs either affecting vitamin D synthesis or metabolism as plausible instruments for MR analyses. We observed some residual co-variation by lifestyle/dietary factors in relation to selected instruments, for example the *GC* SNP was associated with oily fish consumption. The *GC* SNP was also marginally stronger in terms of F-statistic and variation of 25(OH)D concentrations explained than the combined un-weighted allelic scores. Our analyses suggested that use of combined allelic scores reduced confounding since the scores were not associated with lifestyle/dietary factors. The associations between selected SNPs and geographical region, and to some extent with lifestyle/dietary factors, may also indicate a potential issue with regional variation/ancestry. Hence, it is important to consider population stratification in the context of MR studies on vitamin D, and to correct for geographical region/population stratification in related analyses. As genome-wide data becomes more readily available, one approach might be to correct for population stratification using principal components [41]. However, further work is required to demonstrate the extent to which genes expressed in the vitamin D pathway are under genetic selection related to geographical region.

The five genes used in the allelic scores have an important role in the vitamin D metabolic pathway. *DHCR7* and *CYP2R1* function upstream of the 25(OH)D production (synthesis), while *GC*, *CYP24A1* and *CYP27B1* function downstream of the 25(OH)D production (metabolism) (**Figure 2**). *CYP2R1* encodes the enzyme that catalyzes the 25-hydroxylation step in the liver leading to the synthesis of 25(OH)D [42], and as such, it is conceptually the best instrument for MR studies on vitamin D. The SNP included in these analyses is not functional, but it was chosen as it showed the strongest association with 25(OH)D in the published GWAs meta-analyses [23]. In the same study, *DHCR7* was identified as a novel locus for association with vitamin D status [23]. *DHCR7* encodes the enzyme 7-dehydrocholesterol reductase, which converts 7-dehydrocholesterol (7-DHC) to cholesterol, removing the compound from the pathway of vitamin D and onto becoming 25(OH)D. In our study, *DHCR7* was not associated with cholesterol (or other lipid makers) and there was no evidence for effect modification by it on the association of 25(OH)D with cholesterol biomarkers. Also the large GWA meta-analyses on lipid traits failed to identify *DHCR7* as a genetic influence on cholesterol [43], suggesting it has a primary role for vitamin D rather than cholesterol metabolism. Nevertheless, given the possibility for pleiotropic associations, MR studies using *DHCR7* to index 25(OH)D should be interpreted with caution if the suggested association is not also seen for *CYP2R1*.

The strongest of the metabolism markers was vitamin D binding protein (*DBP*), also known as group specific component (Gc), which is involved in the transport of vitamin D and its metabolites [44]. The enzyme encoded by the *CYP24A1* gene plays a crucial role in calcium homeostasis and the vitamin D endocrine system, acting at the first stage of 25(OH)D and 1,25(OH)2D catabolism [45]. *CYP27B1* gene is a well-known candidate for vitamin D pathway [45], as it encodes 1α-hydroxylase, the enzyme that converts 25(OH)D into 1α-25(OH)_2_D (the active hormonal form). Although our findings showed an association of the *CYP27B1* SNP (rs10877012) with 25(OH)D levels just outside the Bonferroni corrected *P*<0.008, we also evaluated rs10877012 as a component of the allele score analysis based on the previous evidence for replication [37]. Conversely, a candidate gene (*OCA2*) based on the association with skin coloration was included in our initial investigations. However, associations between *OCA2* and 25(OH)D have not consistently been observed [27]. In our study evidence for an association between the *OCA2* genotype and 25(OH)D concentrations was abolished by adjustment for lifestyle and social indicators, suggesting that this marker (despite biological plausibility) is not suitable for the use as an instrument in MR studies on vitamin D.

The position of the target gene in the metabolic vitamin D pathway affects the quantification of the expected direction of the SNP-25(OH)D association, which led us to divide the SNPs into those affecting synthesis and metabolism when creating combined allele score indicators. The ability to estimate the magnitude of a possible causal effect for an environmental exposure that can be improved is a key strength with the MR approach [11]. Conceptually, the quantification of the 25(OH)D association for the synthesis markers appears quite straightforward as *DHCR7* and *CYP2R1* contribute to the production of 25(OH)D. Associations are likely to be more complex for the metabolism markers which are involved in the clearance or transport of 25(OH)D (and other vitamin D metabolites). The magnitude of the association between metabolism SNPs and 25(OH)D may depend upon current vitamin D status and requirement, and is likely to be under the influence of (unmeasured, potentially unknown) metabolic feed-back loops. For example, there is evidence to indicate that *GC*, the key 25(OH)D carrier protein, is an important determinant of the bioavailability of vitamin D metabolites to key target cells such as monocytes [46], which will influence the use of related genetic variants as instruments in MR analyses. Separating the SNPs by function into the two allele scores gives flexibility to the subsequent MR models, providing the ability to use them singularly as one instrument or together as two instruments, whilst still accounting for the complex associations with 25(OH)D.

An important strength with MR approach is that the limitations for this method are by and large independent of those typical for other types of observational studies. However, as we have shown in this study, there remains a possibility of residual confounding when using single SNPs as genetic proxy markers. The single most important potential confounder was geographical region, which also had a borderline association with the allele score based on 25(OH)D synthesis SNPs. These analyses highlight the importance of considering population structure/regional variations in MR studies for vitamin D, which given the strong influence of sunlight induced synthesis on serum concentrations, may be particularly vulnerable to this source of confounding.

MR relies on the assumption that genetic variants used as instruments are uncorrelated with other variants that are associated with the outcome outside of the exposure pathway [12]. This assumption may be violated due to the presence of linkage disequilibrium, where variants which are located close to each other are inherited together. It is also possible that the synthesis or metabolism SNPs could have led to biological adaptations during the development (i.e. canalisation) [14]. It is also possible that SNPs used as instruments could have pleiotropic effects where they influence other metabolic pathways independently of the influence on 25(OH)D concentrations. In this study, we found no evidence for strong associations between the SNPs of interest with a range of biomarkers, suggesting specificity for their association with 25(OH)D. An important methodological limitation for MR analyses, including those done in the context of vitamin D, relates to the requirement of very large sample sizes. In the illustrative power calculations included in this paper, we showed how even by a combination of two SNPs into an allele score we were able to half the sample size required. In the MR analysis as modelled by IV regression using a two-stage-least-squares estimator, the association can be biased when both the variance explained by the instruments (in the intermediate phenotype) and the sample size are small [33], [34]. As indicated by the large F-statistics for all allele scores, none of the composite instruments were deemed to be “weak” in our study [33]. However, given the small amount of variation in 25(OH)D explained by these genetic variants, it is clear that the application of MR analyses in the context of vitamin D is resource intensive and as shown here, ∼80,000 participants would be required to detect expected influences on blood pressure using the synthesis SNPs. If both allele scores are combined, the sample size requirement is reduced to ∼40,000 individuals. However, as noted above, quantification of the association for the metabolism SNPs is difficult, and related power calculations will not be correct if there are variations in 25(OH)D bioavailability by GC genotypes as has been suggested [46].

There is great promise in the use of genetic variants as instruments for modifiable exposures, given their ability to avoid some of the limitations of observational epidemiology in making causal inferences. At a public health level the benefits with the approach are evident, as MR studies can be used to imply reductions in disease risk that can be achieved by improving vitamin D status (which in turn, can be done for example by the use of vitamin D supplementation). However, in this paper we demonstrate the complexities of using MR in the context of vitamin D research, most notably the requirement of very large samples, possibility for pleiotropic effects, and the potential of confounding by population stratification. Informative MR studies on vitamin D are likely to be feasible in the context of large international consortia, with the issues on population structure duly considered at the analysis stage. However, within that type of context, MR is highly likely to serve as a useful first-stage approach to testing causality between vitamin D and various health outcomes.

## Supporting Information

Table S1 Call rates and *P* values for Tests of Hardy-Weinberg Equilibrium for Vitamin D Polymorphisms Identified from Candidate Gene and Genome-wide Association Studies.
(DOC)

Table S2 The SNP association with biomarkers adjusted for 25(OH)D, sex and region.
(DOC)

Appendix S1 Sample size calculations.
(DOC)
