# Supplementary material for: Evaluation of Genetic Markers as Instruments for Mendelian Randomization Studies on Vitamin D
Source: PLoS One. 2012 May 21;7(5):e37465. doi: 10.1371/journal.pone.0037465 (PMC3357436; doi:10.1371/journal.pone.0037465)
Supplement: Table S1 — Call rates and P values for Tests of Hardy-Weinberg Equilibrium for Vitamin D Polymorphisms Identified from Candidate Gene and Genome-wide Association Studies. (DOC) [file pone.0037465.s001.doc]

**Table S1. Call rates and *P* values for Tests of Hardy-Weinberg Equilibrium for Vitamin D Polymorphisms Identified from Candidate Gene and Genome-wide Association Studies**

|  | **Gene symbol** | **Chromosomeposition:** | **Gene names** | **SNP** | **Call rate (%)** | **HWE *P* value** |  | |
| --- | --- | --- | --- | --- | --- | --- | --- | --- |
| ***GWA on 25(OH)D*** | | | | | | | |  |
|  | *GC* | 4q12-q13 | Vitamin D binding protein | rs4588^*^ | 83.4 | 0.24 |  | |
|  | *DHCR7/ NADSYN1* | 11q13.4 | 7-dehydrocholesterol reductase/ NAD synthetase 1 | rs12785878^†^ | 100 | 0.80 |  | |
|  | *CYP2R1* | 11p15.2 | Cytochrome P450, family 2, subfamily R, polypeptide 1 | rs10741657^†^ | 82.5 | 0.39 |  | |
|  | *CYP24A1* | 20q13 | Cytochrome P450, family 24, subfamily A, polypeptide 1 | rs6013897^†^ | 98.9 | 0.64 |  | |
| ***Vitamin D pathway genes*** | | | | | | | | |
|  | *CYP27B1* | 12q13.1-q13.3 | Cytochrome P450, family 27, subfamily B, polypeptide 1 | rs10877012 | 95.1 | 0.17 |  | |
|  | *CYP27A1* | 2q33 | Cytochrome P450, family 27, subfamily A, polypeptide 1 | rs17470271 | 80.6 | 0.98 |  | |
| ***GWA on skin colour/ tanning*** | | | | | | | | |
|  | *OCA2* | 15q | Oculocutaneous albinism II | rs7495174 | 96.9 | 0.42 |  | |
|  | *OCA2* | 15q | Oculocutaneous albinism II | rs4778241 | 95.9 | 0.48 |  | |
|  | *OCA2* | 15q | Oculocutaneous albinism II | rs4778138 | 97.4 | 0.09 |  | |
|  | *OCA2* | 15q | Oculocutaneous albinism II | rs12913832^‡^ | 99.9 | 0.72 |  | |
|  | *SLC45A2* | 5p13.2 | Solute carrier family 45, member 2 | rs13289 | 97.4 | 0.90 |  | |
|  | *SLC45A2* | 5q13.2 | Solute carrier family 45, member 2 | rs16891982^‡^ | 97.1 | 0.14 |  | |
|  | *MC1R* | 16q24.3 | Melanocortin 1 receptor | rs11648785^‡^ | 96.5 | 0.18 |  | |
|  | *MC1R* | 16q24.3 | Melanocortin 1 receptor | rs1805005 | 98.4 | 0.27 |  | |
|  | *MC1R* | 16q24.3 | Melanocortin 1 receptor | rs464349^‡^ | 99.5 | 0.05 |  | |
|  | *MC1R* | 16q24.3 | Melanocortin 1 receptor | rs2228479 | 98.3 | 0.26 |  | |
|  | *MC1R* | 16q24.3 | Melanocortin 1 receptor | rs1805007 ^‡^ | 91.6 | 0.17 |  | |
|  | *IRF4* | 6p23-p25 | Interferon regulatory factor 4 | rs12203592^‡^ | 99.8 | 0.01 |  | |
|  | *IRF4* | 6p23-p25 | Interferon regulatory factor 4 | rs12210050^‡^ | 99.9 | 0.73 |  | |
|  | *TYR* | 11q14-q21 | Tyrosinase | rs1393350^‡^ | 100 | 0.50 |  | |

HWE, Hardy-Weinberg equilibrium *P* value; MAF, minor allele frequency

^*^SNP rs4588 used as a proxy for the GWAS SNP rs2282679 (r^2^= 0.98)

^†^SNPs identified from genome-wide association study (GWAS)

^‡^SNPs are from gene-chips and QC procedures are outlined under Genotyping in the Methods section

|  |  |  |  | |  |  |
| --- | --- | --- | --- | --- | --- | --- |
|  | | | | | |  |
|  |  |  |  | |  |  |
|  |  |  |  | |  |  |
|  |  |  |  | |  |  |
|  |  |  |  | |  |  |
|  |  |  |  | |  |  |
|  |  |  |  | |  |  |
|  |  |  |  | |  |  |
|  |  |  |  | |  |  |
|  |  |  |  | |  |  |
|  |  |  |  | |  |  |
|  |  |  |  | |  |  |
|  |  |  |  | |  |  |
|  |  |  |  | |  |  |
|  |  |  |  | |  |  |
|  |  |  |  | |  |  |
|  |  |  |  | |  |  |
|  |  |  |  | |  |  |
|  |  |  |  | |  |  |
|  |  |  |  | |  |  |
|  |  |  |  | |  |  |
|  |  |  |  | |  |  |
|  |  |  |  | |  |  |
|  |  |  |  | |  |  |
|  |  |  |  | |  |  |
|  |  |  |  | |  |  |
|  |  |  |  | |  |  |
|  |  |  |  | |  |  |
|  |  |  |  | |  |  |
|  |  |  |  | |  |  |
|  |  |  |  | |  |  |
|  |  |  |  | |  |  |
|  |  |  |  | |  |  |
|  | | | | | |  |
|  |  |  |  |  | |  |
|  |  |  |  |  | |  |
|  |  |  |  |  | |  |
|  |  |  |  |  | |  |
|  |  |  |  |  | |  |
|  |  |  |  |  | |  |
|  |  |  |  |  | |  |
|  |  |  |  |  | |  |
|  | | | | | |  |
|  |  |  |  | |  |  |
|  |  |  |  | |  |  |
|  |  |  |  | |  |  |
|  |  |  |  | |  |  |
|  |  |  |  | |  |  |
|  |  |  |  | |  |  |
|  |  |  |  | |  |  |
|  |  |  |  | |  |  |
|  |  |  |  | |  |  |
|  |  |  |  | |  |  |
|  |  |  |  | |  |  |
|  |  |  |  | |  |  |
|  |  |  |  | |  |  |
|  |  |  |  | |  |  |
|  |  |  |  | |  |  |
|  |  |  |  | |  |  |
|  |  |  |  | |  |  |
|  |  |  |  | |  |  |
|  |  |  |  | |  |  |
|  |  |  |  | |  |  |
|  |  |  |  | |  |  |
|  |  |  |  | |  |  |
|  |  |  |  | |  |  |
|  |  |  |  | |  |  |
|  |  |  |  | |  |  |
|  |  |  |  | |  |  |
|  |  |  |  | |  |  |
|  |  |  |  | |  |  |
|  |  |  |  | |  |  |
|  |  |  |  | |  |  |
|  |  |  |  | |  |  |
|  |  |  |  | |  |  |
|  | | | | | |  |
|  |  |  |  | |  |  |
|  |  |  |  | |  |  |
|  |  |  |  | |  |  |
|  |  |  |  | |  |  |
|  |  |  |  | |  |  |
|  |  |  |  | |  |  |
|  |  |  |  | |  |  |
|  |  |  |  | |  |  |
|  | | | | | |  |
|  |  |  |  | |  |  |
|  |  |  |  | |  |  |
|  |  |  |  | |  |  |
|  |  |  |  | |  |  |
|  |  |  |  | |  |  |
|  |  |  |  | |  |  |
|  |  |  |  | |  |  |
|  |  |  |  | |  |  |
|  |  |  |  | |  |  |
|  |  |  |  | |  |  |
|  |  |  |  | |  |  |
|  |  |  |  | |  |  |
|  |  |  |  | |  |  |
|  |  |  |  | |  |  |
|  |  |  |  | |  |  |
|  |  |  |  | |  |  |
|  |  |  |  | |  |  |
|  |  |  |  | |  |  |
|  |  |  |  | |  |  |
|  |  |  |  | |  |  |
|  |  |  |  | |  |  |
|  |  |  |  | |  |  |
|  |  |  |  | |  |  |
|  |  |  |  | |  |  |
|  |  |  |  | |  |  |
|  |  |  |  | |  |  |
|  |  |  |  | |  |  |
|  |  |  |  | |  |  |
|  |  |  |  | |  |  |
|  |  |  |  | |  |  |
|  |  |  |  | |  |  |
|  |  |  |  | |  |  |
|  |  |  |  | |  |  |
|  |  |  |  | |  |  |
|  |  |  |  | |  |  |
|  |  |  |  | |  |  |
|  |  |  |  | |  |  |
|  |  |  |  | |  |  |
|  |  |  |  | |  |  |
|  |  |  |  | |  |  |
