# Supplementary material for: Evaluation of Genetic Markers as Instruments for Mendelian Randomization Studies on Vitamin D
Source: PLoS One. 2012 May 21;7(5):e37465. doi: 10.1371/journal.pone.0037465 (PMC3357436; doi:10.1371/journal.pone.0037465)
Supplement: Table S2 — The SNP association with biomarkers adjusted for 25(OH)D, sex and region. (DOC) [file pone.0037465.s002.doc]

**Table S2. The SNP association with biomarkers adjusted for 25(OH)D, sex and region**

| **Biomarker**^*^ | **Gene/Count** | **SNP** | **Coefficient (95% CI)** | | ***P*-value^†^** | **Interaction *P*-value^†^** |
| --- | --- | --- | --- | --- | --- | --- |
| **Coagulation markers** | | | | | |  |
| **vWF** | *DHCR7* | rs12785878 | -0.008 (-0.022, 0.007) | | 0.30 | 0.88 |
|  | *CYP2R1* | rs10741657 | 0.011 (-0.002, 0.023) | | 0.10 | 0.16 |
|  | Synthesis count |  | 0.005 (-0.005, 0.015) | | 0.32 | 0.72 |
|  | *CYP27B1* | rs10877012 | 0.005 (-0.007, 0.017) | | 0.44 | 0.89 |
|  | *CYP24A1* | rs6013897 | 0.000 (-0.015, 0.014) | | 0.97 | 0.88 |
|  | *GC* | rs4588 | -0.005 (-0.019, 0.008) | | 0.43 | 0.33 |
|  | Metabolism count |  | 0.001 (-0.008, 0.009) | | 0.89 | 0.37 |
|  | Metabolism^GWA^ |  | -0.004 (-0.014, 0.007 ) | | 0.50 | 0.80 |
| **tPA** | *DHCR7* | rs12785878 | -0.016 (-0.038, 0.007) | | 0.17 | 0.009(0.14) |
|  | *CYP2R1* | rs10741657 | -0.009 (-0.029, 0.010) | | 0.34 | 0.32 |
|  | Synthesis count |  | -0.012 (-0.028, 0.003) | | 0.13 | 0.13 |
|  | *CYP27B1* | rs10877012 | -0.009 (-0.028, 0.01) | | 0.38 | 0.46 |
|  | *CYP24A1* | rs6013897 | -0.013 (-0.036, 0.01) | | 0.27 | 0.075 |
|  | *GC* | rs4588 | 0.007 (-0.014, 0.028) | | 0.51 | 0.34 |
|  | Metabolism count |  | -0.001 (-0.014, 0.012) | | 0.88 | 0.04(0.51) |
|  | Metabolism^GWA^ |  | 0.004 (-0.020, 0.012) | | 0.60 | 0.55 |
| **D-dimer** | *DHCR7* | rs12785878 | 0.005 (-0.018, 0.028) | | 0.66 | 0.40 |
|  | *CYP2R1* | rs10741657 | 0.005 (-0.016, 0.025) | | 0.65 | 0.005(0.08) |
|  | Synthesis count |  | 0.006 (-0.01, 0.022) | | 0.48 | 0.25 |
|  | *CYP27B1* | rs10877012 | 0.007 (-0.012, 0.026) | | 0.48 | 0.91 |
|  | *CYP24A1* | rs6013897 | -0.006 (-0.03, 0.018) | | 0.63 | 0.64 |
|  | *GC* | rs4588 | 0.003 (-0.019, 0.025) | | 0.80 | 0.79 |
|  | Metabolism count |  | 0.003 (-0.010, 0.017) | | 0.65 | 0.37 |
|  | Metabolism^GWA^ |  | -0.001(-0.017, 0.016) | | 0.95 | 0.80 |
| **Fibrinogen** | *DHCR7* | rs12785878 | 0.001 (-0.008, 0.009) | | 0.90 | 0.42 |
|  | *CYP2R1* | rs10741657 | 0.003 (-0.004, 0.011) | | 0.40 | 0.28 |
|  | Synthesis count |  | 0.001 (-0.005, 0.007) | | 0.83 | 0.81 |
|  | *CYP27B1* | rs10877012 | 0.002 (-0.005, 0.009) | | 0.57 | 0.43 |
|  | *CYP24A1* | rs6013897 | -0.002 (-0.011, 0.007) | | 0.61 | 0.72 |
|  | *GC* | rs4588 | -0.002 (-0.010, 0.006) | | 0.66 | 0.60 |
|  | Metabolism count |  | -0.001 (-0.006, 0.004) | | 0.74 | 0.71 |
|  | Metabolism^GWA^ |  | -0.003 (-0.009, 0.003) | | 0.37 | 0.65 |
| **Inflammatory marker** | | | | | |  |
| **CRP** | *DHCR7* | rs12785878 | 0.020 (-0.031, 0.071) | 0.44 | | 0.40 |
|  | *CYP2R1* | rs10741657 | 0.000 (-0.045, 0.045) | 0.99 | | 0.07 |
|  | Synthesis count |  | 0.004 (-0.032, 0.039) | 0.83 | | 0.26 |
|  | *CYP27B1* | rs10877012 | 0.017 (-0.026, 0.06) | 0.44 | | 0.87 |
|  | *CYP24A1* | rs6013897 | -0.046 (-0.098, 0.006) | 0.08 | | 0.78 |
|  | *GC* | rs4588 | -0.051 (-0.099, -0.003) | 0.04 | | 0.96 |
|  | Metabolism count |  | -0.025 (-0.055, 0.005) | 0.10 | | 0.34 |
|  | Metabolism^GWA^ |  | -0.051 (-0.088, -0.015) | 0.006(0.09) | | 0.99 |
| **Lipid markers** | | | | | |  |
| **Triglycerides** | *DHCR7* | rs12785878 | -0.010 (-0.033, 0.013) | | 0.38 | 0.94 |
|  | *CYP2R1* | rs10741657 | 0.008 (-0.013, 0.028) | | 0.47 | 0.14 |
|  | Synthesis count |  | 0.000 (-0.016, 0.017) | | 0.97 | 0.43 |
|  | *CYP27B1* | rs10877012 | 0.007 (-0.013, 0.027) | | 0.48 | 0.18 |
|  | *CYP24A1* | rs6013897 | 0.007 (-0.017, 0.031) | | 0.54 | 0.22 |
|  | *GC* | rs4588 | -0.003 (-0.025, 0.019) | | 0.81 | 0.92 |
|  | Metabolism count |  | 0.006 (-0.008, 0.019) | | 0.42 | 0.22 |
|  | Metabolism^GWA^ |  | 0.000 (-0.017, 0.017) | | 0.96 | 0.49 |
| **LDL** | *DHCR7* | rs12785878 | -0.011 (-0.022, 0.001) | | 0.07 | 0.61 |
|  | *CYP2R1* | rs10741657 | -0.006 (-0.016, 0.005) | | 0.28 | 0.80 |
|  | Synthesis count |  | -0.007 (-0.015, 0.001) | | 0.08 | 0.76 |
|  | *CYP27B1* | rs10877012 | 0.004 (-0.006, 0.013) | | 0.47 | 0.41 |
|  | *CYP24A1* | rs6013897 | -0.003 (-0.015, 0.009) | | 0.62 | 0.07 |
|  | *GC* | rs4588 | -0.004 (-0.015, 0.007) | | 0.50 | 0.89 |
|  | Metabolism count |  | 0.001 (-0.006, 0.008) | | 0.82 | 0.48 |
|  | Metabolism^GWA^ |  | -0.003 (-0.011, 0.005) | | 0.48 | 0.56 |
| **HDL** | *DHCR7* | rs12785878 | -0.002 (-0.012, 0.007) | | 0.62 | 0.13 |
|  | *CYP2R1* | rs10741657 | 0.000 (-0.008, 0.009) | | 0.95 | 0.23 |
|  | Synthesis count |  | -0.002 (-0.008, 0.005) | | 0.63 | 0.39 |
|  | *CYP27B1* | rs10877012 | 0.001 (-0.007, 0.009) | | 0.77 | 0.15 |
|  | *CYP24A1* | rs6013897 | -0.003 (-0.013, 0.007) | | 0.52 | 0.27 |
|  | *GC* | rs4588 | 0.000 (-0.010, 0.009) | | 0.94 | 0.33 |
|  | Metabolism count |  | -0.002 (-0.007, 0.004) | | 0.55 | 0.31 |
|  | Metabolism^GWA^ |  | -0.001 (-0.008, 0.007) | | 0.88 | 0.17 |
| **Cholesterol** | *DHCR7* | rs12785878 | -0.006 (-0.014, 0.001) | | 0.10 | 0.26 |
|  | *CYP2R1* | rs10741657 | -0.003 (-0.009, 0.004) | | 0.39 | 0.90 |
|  | Synthesis count |  | -0.005 (-0.01, 0.001) | | 0.082 | 0.64 |
|  | *CYP27B1* | rs10877012 | 0.003 (-0.003, 0.009) | | 0.37 | 0.53 |
|  | *CYP24A1* | rs6013897 | -0.002 (-0.009, 0.006) | | 0.70 | 0.15 |
|  | *GC* | rs4588 | -0.001 (-0.008, 0.006) | | 0.73 | 0.70 |
|  | Metabolism count |  | 0.001 (-0.004, 0.005) | | 0.71 | 0.54 |
|  | Metabolism^GWA^ |  | 0.001 (-0.007, 0.004) | | 0.65 | 0.56 |
| **Lung function marker** | | | | | |  |
| **FEV** | *DHCR7* | rs12785878 | 0.001 (-0.023, 0.025) | | 0.95 | 0.04 (0.54) |
|  | *CYP2R1* | rs10741657 | 0.014 (-0.007, 0.035) | | 0.20 | 0.44 |
|  | Synthesis count |  | 0.010 (-0.007, 0.027) | | 0.23 | 0.73 |
|  | *CYP27B1* | rs10877012 | 0.001 (-0.02, 0.021) | | 0.95 | 0.70 |
|  | *CYP24A1* | rs6013897 | 0.004 (-0.021, 0.029) | | 0.76 | 0.97 |
|  | *GC* | rs4588 | 0.007 (-0.016, 0.03) | | 0.56 | 0.51 |
|  | Metabolism count |  | 0.006 (-0.008, 0.02) | | 0.43 | 0.77 |
|  | Metabolism^GWA^ |  | -0.006 (-0.011, 0.024) | | 0.47 | 0.03 (0.48) |
| **Cardiovascular disease related markers** | | | | | |  |
| **Diastolic BP** | *DHCR7* | rs12785878 | 0.003 (-0.003, 0.008) | | 0.34 | 0.68 |
|  | *CYP2R1* | rs10741657 | -0.004 (-0.008, 0.001) | | 0.12 | 0.04 (0.62) |
|  | Synthesis count |  | -0.002 (-0.006, 0.002) | | 0.29 | 0.46 |
|  | *CYP27B1* | rs10877012 | -0.002 (-0.006, 0.003) | | 0.50 | 0.11 |
|  | *CYP24A1* | rs6013897 | -0.005 (-0.011, 0.000) | | 0.065 | 0.35 |
|  | *GC* | rs4588 | -0.004 (-0.009, 0.001) | | 0.17 | 0.004 (0.07) |
|  | Metabolism count |  | -0.003 (-0.006, 0) | | 0.09 | 0.69 |
|  | Metabolism^GWA^ |  | -0.004 (-0.008, 0.000) | | 0.03 (0.50) | 0.33 |
| **Systolic BP** | *DHCR7* | rs12785878 | 0.003 (-0.002, 0.007) | | 0.30 | 0.57 |
|  | *CYP2R1* | rs10741657 | 0.000 (-0.004, 0.005) | | 0.89 | 0.072 |
|  | Synthesis count |  | 0.001 (-0.003, 0.004) | | 0.67 | 0.52 |
|  | *CYP27B1* | rs10877012 | -0.001 (-0.005, 0.003) | | 0.67 | 0.49 |
|  | *CYP24A1* | rs6013897 | -0.005 (-0.01, 0) | | 0.03 | 0.38 |
|  | *GC* | rs4588 | -0.003 (-0.007, 0.002) | | 0.26 | 0.058 |
|  | Metabolism count |  | -0.002 (-0.005, 0.001) | | 0.15 | 0.77 |
|  | Metabolism^GWA^ |  | -0.004 (-0.008, 0.000) | | 0.03(0.39) | 0.16 |
| **IgE** | *DHCR7* | rs12785878 | -0.010 (-0.069, 0.05) | | 0.75 | 0.47 |
|  | *CYP2R1* | rs10741657 | 0.058 (0.005, 0.111) | | 0.03 | 0.39 |
|  | Synthesis count |  | 0.037 (-0.005, 0.079) | | 0.08 | 0.79 |
|  | *CYP27B1* | rs10877012 | 0.047 (-0.003, 0.098) | | 0.07 | 0.72 |
|  | *CYP24A1* | rs6013897 | 0.016 (-0.045, 0.078) | | 0.60 | 0.88 |
|  | *GC* | rs4588 | -0.016 (-0.072, 0.041) | | 0.59 | 0.03 (0.44) |
|  | Metabolism count |  | 0.019 (-0.016, 0.054) | | 0.29 | 0.59 |
|  | Metabolism^GWA^ |  | 0.005 (-0.039, 0.048) | | 0.84 | 0.58 |
| **IGF-1** | *DHCR7* | rs12785878 | 0.001 (-0.012, 0.013) | | 0.90 | 0.091 |
|  | *CYP2R1* | rs10741657 | -0.002 (-0.013, 0.009) | | 0.70 | 0.21 |
|  | Synthesis count |  | -0.001 (-0.010, 0.008) | | 0.77 | 0.99 |
|  | *CYP27B1* | rs10877012 | -0.008 (-0.019, 0.002) | | 0.12 | 0.31 |
|  | *CYP24A1* | rs6013897 | -0.001 (-0.014, 0.012) | | 0.84 | 0.15 |
|  | *GC* | rs4588 | 0.012 (-0.001, 0.024) | | 0.06 | 0.61 |
|  | Metabolism count |  | -0.002 (-0.01, 0.005) | | 0.52 | 0.70 |
|  | Metabolism^GWA^ |  | 0.004 (-0.005, 0.013) | | 0.39 | 0.62 |
| **HbA1c** | *DHCR7* | rs12785878 | 0.000 (-0.004, 0.005) | | 0.94 | 0.13 |
|  | *CYP2R1* | rs10741657 | 0.000 (-0.004, 0.004) | | 0.90 | 0.63 |
|  | Synthesis count |  | 0.000 (-0.003, 0.003) | | 0.82 | 0.60 |
|  | *CYP27B1* | rs10877012 | 0.000 (-0.003, 0.004) | | 0.88 | 0.39 |
|  | *CYP24A1* | rs6013897 | 0.001 (-0.004, 0.005) | | 0.72 | 0.69 |
|  | *GC* | rs4588 | -0.001 (-0.005, 0.003) | | 0.56 | 0.46 |
|  | Metabolism count |  | 0.000 (-0.002, 0.003) | | 0.84 | 0.17 |
|  | Metabolism^GWA^ |  | 0.000 (-0.003, 0.003) | | 0.90 | 0.51 |

^*^Where required the biomarker has been natural log transformed to achieve normal distribution

^†^In brackets, the *p*-value adjusted for multiple testing.
