# Supplementary material for: Evaluation of Genetic Markers as Instruments for Mendelian Randomization Studies on Vitamin D
Source: PLoS One. 2012 May 21;7(5):e37465. doi: 10.1371/journal.pone.0037465 (PMC3357436; doi:10.1371/journal.pone.0037465)
Supplement: Appendix S1 — Sample size calculations. (DOC) [file pone.0037465.s003.doc]

**Appendix S1 – Sample size calculations**

Mendelian randomisation (MR) studies can be modelled using two-stage least squares (2SLS) regression for instrumental variable (IV) analysis. In this setting the endogenous variable is environmental exposure (in our example 25(OH)D concentrations), exogenous /instrumental variable is the genetic marker (in our example SNPs or allele count from multiple SNPs), and outcome is a quantitative bio-marker (in our example the standardized systolic BP). For 2SLS regression there is no standard approach for estimating power and sample size, however one way is through repetitive simulation of a dataset. This was done by simulating a dataset of a fixed sample size (*n*) by imposing constraints on the known relationships, running the regression model and then testing the parameter of interest at a fixed significant level *α*. The dataset was re-simulated a 1000 times for the same sample size (*n*) and the proportion of times the parameter of interest was less than the significant level *α* was used to indicate the power of the study with the sample size in question [1].

To create a dataset for the simulation, the models are defined as:

$\begin{matrix} x_{i}=\alpha_{0}+\alpha_{1}g_{i}+\varepsilon_{xi} \\ y_{i}=\beta_{0}+\beta_{1}x_{i}+\varepsilon_{yi} \end{matrix}$,

where *i* is the number of individuals, *x_i_,g_i_, y_i_* are the exposure, genetic variant, and outcome for each individual, respectively, with $\varepsilon_{xi}\sim N\left(0,\sigma_{1}^{2}\right)\text{ and }\varepsilon_{yi}\sim N\left(0,\sigma_{2}^{2}\right)$. The genetic variant *g_i_* takes the values for the single marker $\left\{0,1,2\right\}$ and joint allele count $\left\{0,1,2,3\right\}$. We assumed an effect of a 5% reduction in a standardized blood pressure outcome by 10nmol/l increase in 25(OH)D based on effect estimates from the 1958BC. The MAF of the SNPs were also based on the proportions from the 1958BC.

Reference List

1. Feiveson AH (2002) Power by simulation. Stata Journal 2: 107-124
